# Characterisation of the heat shock protein Tid and its involvement in stress response regulation in *Apis cerana*

**DOI:** 10.3389/fphys.2022.1068873

**Published:** 2022-12-22

**Authors:** Guilin Li, Chenghao Zhang, Hongfang Wang, Wenli Xia, Xinyi Zhang, Zhenguo Liu, Ying Wang, Hang Zhao, Baohua Xu

**Affiliations:** ^1^ College of Life Sciences, Qufu Normal University, Qufu, China; ^2^ College of Animal Science and Technology, Shandong Agricultural University, Taian, Shandong, China

**Keywords:** Tid, heat shock protein, honey bees, stress response, antioxidant genes

## Abstract

**Objective:** The impact of various environmental stresses on native *Apis cerana cerana* fitness has attracted intense attention in China. However, the defence responses of *A. cerana cerana* to different stressors are poorly understood. Here, we aimed to elucidate the regulatory mechanism mediated by the tumorous imaginal discs (Tid) protein of *A. cerana cerana* (AccTid) in response to stressors.

**Methods:** We used some bioinformatics softwares to analyse the characterisation of Tid. Then, qRT–PCR, RNA interference and heat resistance detection assays were used to explore the function of Tid in stress response in *A. cerana cerana*.

**Results:** *AccTid* is a homologous gene of human Tid1 and *Drosophila* Tid56, contains a conserved J domain and belongs to the heat shock protein DnaJA subfamily. The level of *AccTid* induced expression was increased under temperature increases from 40°C to 43°C and 46°C, and *AccTid* knockdown decreased the heat resistance of *A. cerana cerana*, indicating that the upregulation of *AccTid* plays an important role when *A. cerana cerana* is exposed to heat stress. Interestingly, contrary to the results of heat stress treatment, the transcriptional level of *AccTid* was inhibited by cold, H_2_O_2_ and some agrochemical stresses and showed no significant change under ultraviolet ray and sodium arsenite stress. These results suggested that the requirement of *A. cerana cerana* for Tid differs markedly under different stress conditions. In addition, knockdown of *AccTid* increased the mRNA levels of some Hsps and antioxidant genes. The upregulation of these Hsps and antioxidant genes may be a functional complement of *AccTid* knockdown.

**Conclusion:** *AccTid* plays a crucial role in *A. cerana cerana* stress responses and may mediate oxidative damage caused by various stresses. Our findings will offer fundamental knowledge for further investigations of the defence mechanism of *A. cerana cerana* against environmental stresses.

## Introduction

Honeybees are prominent pollinators that provide crucial ecosystem services by pollinating many crops and wild plants; however, in doing so, they are subjected to many environmental stresses, such as heat, cold, ultraviolet rays (UV) and a variety of agrochemicals. These stress factors are detrimental to the fitness of honeybees and are the main drivers of honeybee colony losses (Potts et al., 2010; Even et al., 2012; Goulson et al., 2015). For example, imidacloprid triggers mortality and activates reactive oxygen species (ROS), probably by inducing Fe^2+^ overload (He et al., 2021a). In honeybees, abamectin is highly toxic (Bai and Ogbourne, 2016), and the death rate of honeybees is increased under abamectin stress conditions (Li et al., 2022a). Beta-cypermethrin has chronic toxic impacts on the larvae of honeybees and decreases the fitness of honeybees at low doses (He et al., 2022). Heat stress has effects on the immunocompetence of drones, workers and queens (Medina et al., 2020). Therefore, it is necessary to explore the underlying molecular defence systems and related key genes of honeybees involved in their adaptation to environmental stresses and protection from different stresses to facilitate the selective and protected breeding of honeybees with enhanced resistance in response to stress factors.

*Apis cerana*, a subspecies of the Asiatic honeybee *Apis cerana*, is an indigenous honeybee species in China and provides major economic benefits to China’s agricultural sector and beekeepers. The pollinating services of *A. cerana* are superior to those of other bee species in certain plants. For example, *A. cerana* outperforms *Apis mellifera* in increasing the pollination of *Pyrus bretschneideri* in China (Gemeda et al., 2017). However, *A. cerana* colony losses have occurred in recent years (Theisen-Jones and Bienefeld, 2016; Chen et al., 2017). Exposure to different environmental stresses is one of the reasons for *A. cerana* colony loss (Even et al., 2012; Goulson et al., 2015), and the defence mechanisms of *A. cerana* against environmental stresses require further study.

The tumorous imaginal discs (Tid) proteins exist in many animals. However, Tid is often referred to as Tid56 (tumour suppressor lethal (2) tumorous imaginal discs) in *Drosophila* and Tid1 in human. Although Tid56 is classified as a tumour suppressor, *Tid56* was the first identified, and only known gene that encodes a protein belonging to the DnaJ cochaperone family (Kurzik-Dumke et al., 1995; Kurzik-Dumke et al., 1998; Schilling et al., 1998). DnaJ (also referred to as heat shock protein 40, Hsp40), has been divided into classes A (DnaJA), B (DnaJB) and C (DnaJC) according to their different domains and motifs, and is functionally related to the Hsp70 family as a cochaperone (Hennessy et al., 2000; Kampinga and Craig, 2010). The structural domains of Tid, Tid56 and Tid1 are well conserved, and all contain the N-terminal signature J domain. In humans, Tid1 is classified into the DnaJA subfamily and known as DnaJA3 (Kurzik-Dumke et al., 1995; Sterrenberg et al., 2011; Patra et al., 2019). These results indicate that Tid, Tid56 and Tid1 belong to the DnaJA subfamily. Furthermore, the human *Tid1* gene encodes two alternatively spliced protein isoforms, Tid1-short (Tid1-S) and Tid1-long (Tid1-L). The difference between Tid1-S and Tid1-L is the length at their carboxy terminal end. Tid1-S and Tid1-L contain 6 and 33 unique amino acids at their carboxy terminal ends, respectively (Lu et al., 2006; Banerjee et al., 2022). Tid1-L and Tid1-S can form heterocomplexes. Tid1-L, rather than Tid1-S, interacts with Hsc70 (belonging to the Hsp70 family), and the carboxyl terminus of Tid1-L determines the interaction between Tid1-L and Hsc70 (Lu et al., 2006).

Functional studies of Tid have mainly focused on Tid1 and Tid56, and significant roles of Tid1 or Tid56 in multiple processes have been established. For example, imaginal discs null mutants of Tid56 lead to lethal tumour development in *Drosophila melanogaster* (Kurzik-Dumke et al., 1992; Kurzik-Dumke et al., 1995). Tid1 participates in agrin signalling regulation, which is important for synaptic development (Linnoila et al., 2008) and is vital in maintaining mitochondrial DNA integrity and membrane potential homogeneity (Ng et al., 2014). The glycosylation of Tid1 plays vital roles in attenuating tumorigenicity and mediating head and neck cancer metastasis (Chen et al., 2018). Tid1 is necessary during skeletal myogenesis, and Tid1 deficiency impairs mitochondrial activity and decreases ATP production in mice (Cheng et al., 2016). A variant of Tid1 is related to a human disease characterised by polyneuropathy and developmental delay (Patra et al., 2019). In addition, it has been proven that Tid1 can mediate cell death, apoptosis and macroautophagy (Syken et al., 1999; Syken et al., 2003; Niu et al., 2015). Tid protein levels are increased after whiteflies are infected by tomato yellow leaf curl virus, and high levels of tomato yellow leaf curl virus are found following the knockdown of *Tid* with double-stranded RNA (dsRNA) (Zhao et al., 2020), indicating that Tid may play an important role in insect infection. However, these studies have mainly focused on *Drosophila melanogaster*, humans and whiteflies. The functions of Tid in many animals remain unclear.

Notably, some *DnaJ* genes are related to stress responses, as shown for *DnaJ-1* in *D. melanogaster*; *DnaJC8*, *DnaJB12* and *DnaJA1* in *A. cerana*; and *Hsp40A4* in *Paralichthys olivaceus* (Dong et al., 2006; Neal et al., 2006; Li et al., 2018; Li et al., 2020)*.* However, whether *Tid*, as a member of the *DnaJ* subfamily, functions in the stress response remains elusive. We aimed to investigate the structural characteristics and response of *Tid* under stress conditions to better understand the molecular basis of *A. cerana* defence against environmental stress. We analysed the structural characteristics of AccTid and explored its evolutionary relationships with other animal Tid proteins. We also investigated the expression pattern of *AccTid* under different stress conditions and used an RNA interference (RNAi) assay to further examine the role of *AccTid*. The results may shed light on the defence response of *A. cerana* to environmental stresses.

## Materials and methods

### Cloning of the coding sequence of *AccTid*

The coding sequence (CDS) of *AccTid* was isolated *via* PCR using specific primers (Table S1) designed according to the CDS of *Tid* from *Apis cerana* (*AcTid*, Gene ID: 107999736). PCR was performed using *TransFast* ^®^ Taq DNA Polymerase (TransGen Biotech, China) following the manufacturer’s protocol.

### Bioinformatics analysis of AccTid

The homologous protein sequences of AccTid in other animals were downloaded from NCBI. The J domains of the Tid proteins of honeybees and other animals were searched against the NCBI Conserved Domain and SMART databases and were described previously (Ohtsuka and Hata, 2000a). The tertiary structure of the AccTid J domain was constructed using SWISS-MODEL and further analysed using SPDBV. The neighbour-joining method in MEGA was used to establish a phylogenetic tree of Tid proteins from different animal species. The neighbour-joining method is a common method for constructing phylogenetic trees *via* the distance method, which is based on the principle of minimum evolution and does not use optimization criteria. DNAMAN was used to perform multiple alignment of amino acid sequences.

### Stress treatment of *A. cerana*

There is a division of labour in honeybees. Labour outside of the colony for the collection of resources, such as nectar and pollen, is usually provided by older workers (18–32-day-old adults) (Behrends et al., 2007; Rueppell et al., 2009; Vance et al., 2009). We sought to explore the role of *AccTid* under different stresses in older workers. Honeybees at 18 days old are just beginning to engage in collection work and are not particularly skilled in collection procedures. In addition, unknown exposure to stressors in all of the older workers when they perform collection work will add variability to the results. To reduce the effect of these factors, we selected 19-day-old honeybees as experimental subjects.

*A. cerana* workers at 19 days of age were obtained from a healthy outdoor colony reared at a site with no agrochemical use at Shandong Agricultural University from May to September. To acquire 19-day-old honeybees, newly emerged workers were marked using paint, and they were collected 19 days later and randomly divided into 17 groups (n = 50/group). After the preliminary experiment, each group of 50 honeybees will ensure that we have enough live honeybee samples for the processing time. For the treatment group, there was a mortality phenomenon during the experiment. If there are any honeybees left after the sample is taken, they will be released. Groups 1–4 were placed in four incubators with temperatures set at 46°C, 43°C, 40°C, and 4°C, respectively. The 46°C, 43°C, and 40°C treatments were considered to impose heat stress, and the 4°C treatment was considered to impose cold stress. Each honeybee in groups 5–10 was fed 1 µL field-realistic doses of beta-cypermethrin, methomyl, abamectin, imidacloprid, spirodiclofen, bifenthrin or paraquat. The manufacturers, active ingredient contents and dosage forms of the above six agrochemicals were presented in Supplementary Table S2. The field-realistic doses of the above six agrochemicals were obtained by dissolution or dilution with water following the manufacturer’s instructions. Group 11–14 was treated with UV (30 mJ/cm^2^), H_2_O_2_ (2 mM), paraquat (1.3 mg/ml), sodium arsenite (0.1 mM). Group 15 was the control for groups 1–14; these honeybees were left untreated and were maintained at 33°C. Group 16 was reared in an incubator whose temperature was set at 40°C, and its control, group 17, was incubated at 33°C. All groups were maintained under darkness at 70% relative humidity and were fed a 30% sucrose solution. The collection time of each group is listed in Table S3, and each collection sample contained 4 *A. cerana*. Three independent experiments were performed for each treatment.

### RNA extraction and cDNA synthesis

RNA extraction was performed using whole *A. cerana* individuals, and each RNA sample was pooled from 4 *A. cerana* individuals. Total RNA was extracted using TRIzol (TaKaRa, China) following the manufacturer’s protocol. The RNA was dissolved in 50 μL of RNase-free water. The RNA was later reverse transcribed into cDNA using HiScript^®^ II Q RT SuperMix for qPCR (+ gDNA Wiper) (TaKaRa, China) based on the manufacturer’s manual.

### Quantitative real-time PCR (qRT–PCR)

A SYBR^®^ Green Premix *Pro Taq* HS qPCR Kit provided by Accurate Biotechnology (Hunan) Co. Ltd. (China) was used to carry out qRT–PCR. The qRT–PCR mixture (20 µl) included .5 µl of each primer (Supplementary Table S1), 1 µl of cDNA, 8 µl of RNase-free water, and 10 µl of 2X SYBR^®^ Green *Pro Taq* HS Premix. The reactions were run on a CFX96 Real-Time Detection System (Bio-Rad, United States). The primers used for qRT–PCR are listed in Supplementary Table S1. The cycling conditions were as follows: 95°C (30 s), then 40 cycles of 5 s at 95°C and 30 s at 60°C, followed by a final melting curve analysis with ramping from 65°C to 95°C in 5°C intervals every 5 s. Gene expression levels were calculated using the 2^−ΔΔCT^ method and normalised to the mRNA level of the internal control gene *β-actin* (Li et al., 2012; Wang et al., 2012). The reference gene *β-actin* has been evaluated to confirm that there was no change during the stress response (Lourenco et al., 2008; Scharlaken et al., 2008; Li et al., 2012; Wang et al., 2012). All the primers used for qRT–PCR were designed according to the principles of qRT–PCR primer design, and each qRT–PCR primer pair has coefficients (*R* ^2^) at .98–1.00, efficiency (E) values at 90%–110% and one single peak melting curve. Each qRT–PCR was carried out with three independent biological replicates, which were three replicates from the exposures, and each cDNA sample was performed in three technical replicates.

### Preparation of double-stranded RNA (dsRNA)

To knock down *Tid* expression in *A. cerana*, dsRNA-mediated gene silencing was used. A fragment (499 bp) of *AccTid* was amplified from *A. cerana* cDNA by PCR using primers (Supplementary Table S1) with the T7 RNA polymerase promoter sequence added at the 5′ end. The PCR products were confirmed by 1% agarose gel electrophoresis and purified using a DNA Extraction Kit (Solarbio, China). The purified PCR product was then used as a template for the synthesis of dsRNA targeting *AccTid* using the T7 RiboMAX™ Express RNAi System (Promega, United States) following the manufacturer’s protocol. A dsRNA targeting fragment (500 bp) of *GFP* (GenBank: U87974.1) was also generated and used as a control. The dsRNA was finally dissolved in nuclease-free water.

### Honeybees for RNAi efficiency validation

The 19-day-old workers were divided into two groups, and each group contained 20 honeybee individuals. We pinched the honeybees’ wings with one hand and then used the other hand to hold a 2.5 µl pipette to feed the honeybees dsRNA. Each honeybee from one group was fed 5 µg of dsRNA-Tid, and each honeybee from the other group (control) was fed 5 µg of dsRNA-GFP. The above two groups were then maintained at 33°C under 24 h of darkness and 70% relative humidity. After feeding for 1 day, the honeybees were collected, and the efficiency of RNAi was determined using qRT–PCR. Three biological replicates were performed in the RNAi assays.

### Heat resistance detection of honeybees after knockdown of *AccTid*

*A. cerana* (19-day-old workers) were divided into 4 groups. Group 1–2 were fed 5 µg dsRNA-Tid, and group 3–4 were fed 5 µg dsRNA-GFP. Then, group 1 and group 3 were exposed to 40°C for heat tolerance experiments, group 2 and group 4 were maintained at 33°C, and the percent survival of each group was calculated every 4 h.

### Detection of the impacts of RNAi *AccTid* on other *Hsps* and antioxidant-related genes

The expression levels of other *Hsps* and antioxidant-related genes were detected using qRT–PCR after the honeybees had been fed dsRNA-AccTid for 1 day, while the control honeybees were fed dsRNA-GFP for 1 day. The primer sequences are presented in Supplementary Table S1. The qRT–PCR primers were referred to other researches (Yan et al., 2012; Yu et al., 2012; Zhang et al., 2012; Shi et al., 2013; Yan et al., 2013; Yao et al., 2013; Zhang et al., 2013; Yan et al., 2014; Yao et al., 2014; Zhang et al., 2014; Huaxia et al., 2015; Liu et al., 2015; Liu et al., 2016; Zhao et al., 2018; Li et al., 2022b) or designed in our study.

### Statistical analysis

Student’s *t*-test was used to carry out statistical analysis between two groups, where ** indicates *p* < .01. One-way ANOVA followed by Bonferroni’s correction was used for statistical analyses between three or more groups, where *p < .05 and **p < .01.

## Results

### Identification of *AccTid*

**FIGURE 1:**
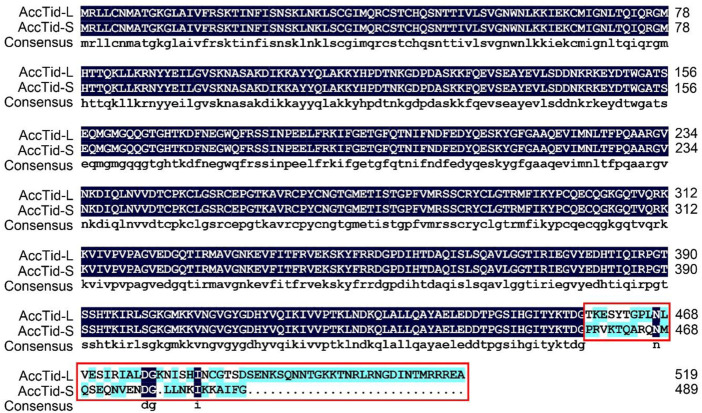
Multiple amino acid alignment of AccTid-L and AccTid-S. The difference between AccTid-L and AccTid-S is indicated with a red box. AccTid-L: long isoform of AccTid. AccTid-S: short isoform of AccTid.

There are two isoforms of *AcTid* deposited in NCBI. We also isolated two isoforms of *AccTid* in this study. The CDS of one isoform of *AccTid* was 1,563 bp and encoded 520 amino acids; we named this isoform *AccTid-long* (*AccTid-L*) (Supplementary Figure S1). The CDS of the other isoform of *AccTid* was 1,471 bp and encoded 489 amino acids; we named this isoform *AccTid-short* (*AccTid-S*) (Supplementary Figure S2). AccTis-L and AccTid-S showed 88.65% homology, and the 457 amino acids at the beginning of AccTis-L and AccTid-S were identical (Figure 1).

### Analysis of the evolutionary relationships of AccTid with other animal tid proteins

**FIGURE 2:**
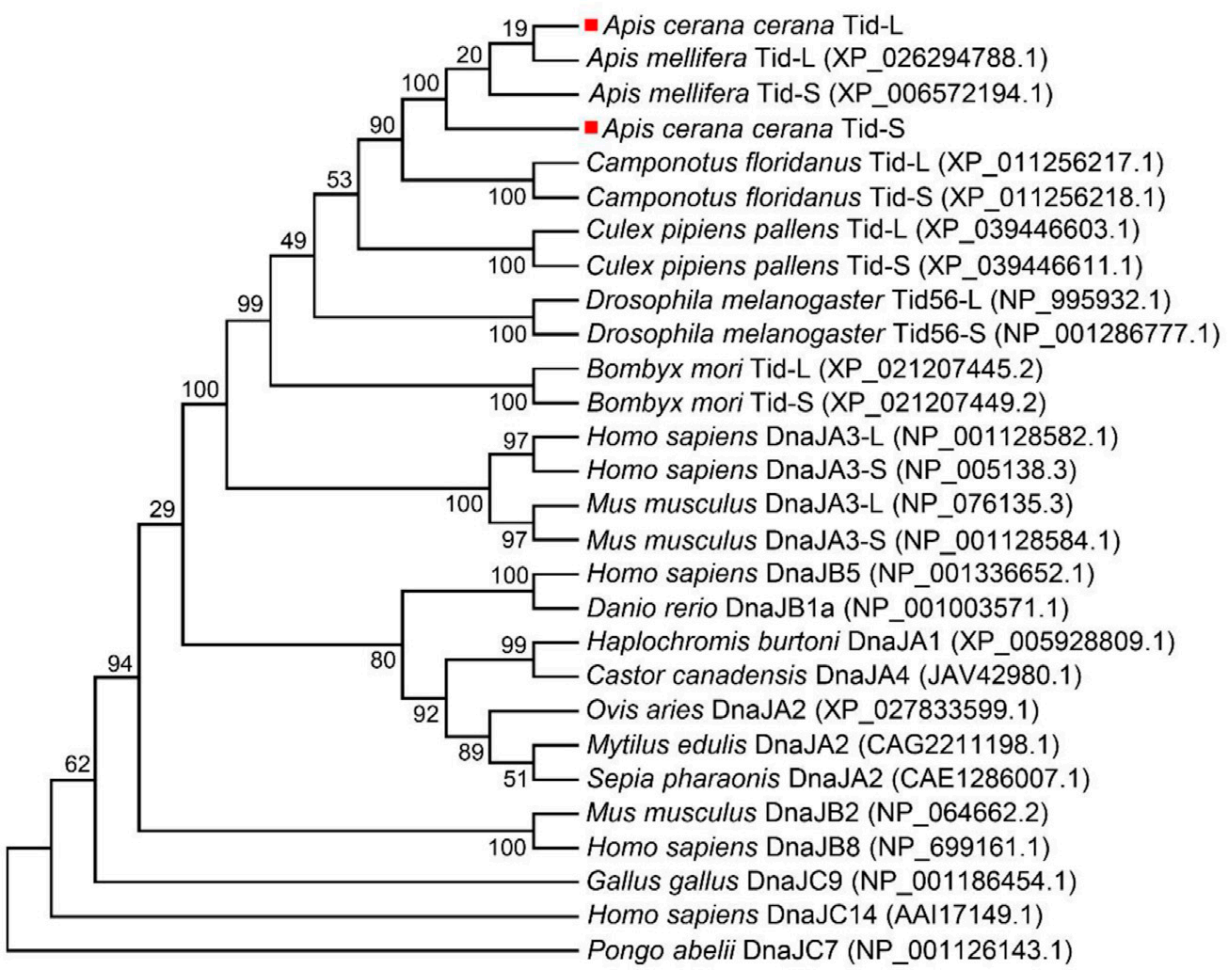
Phylogenetic analysis of Tid proteins from various animals. AccTid-L and AccTid-S are indicated with red squares. -L: long isoform. -S: short isoform.

To investigate the evolutionary relationships of Tid proteins among different animals, we carried out a phylogenetic analysis. In this analysis, we found that Tid, Tid1 and Tid56 were more closely related to DnaJA subfamily proteins than to DnaJB and DnaJC subfamily proteins. Furthermore, the homology of AccTid was closest to that of *A. mellifera* Tid (AmTid) (Figure 2). AmTis-L was grouped with AmTid-S, while AccTid-S was not grouped with AccTid-L (Figure 2).

### Sequence analysis of AccTid

**FIGURE 3:**
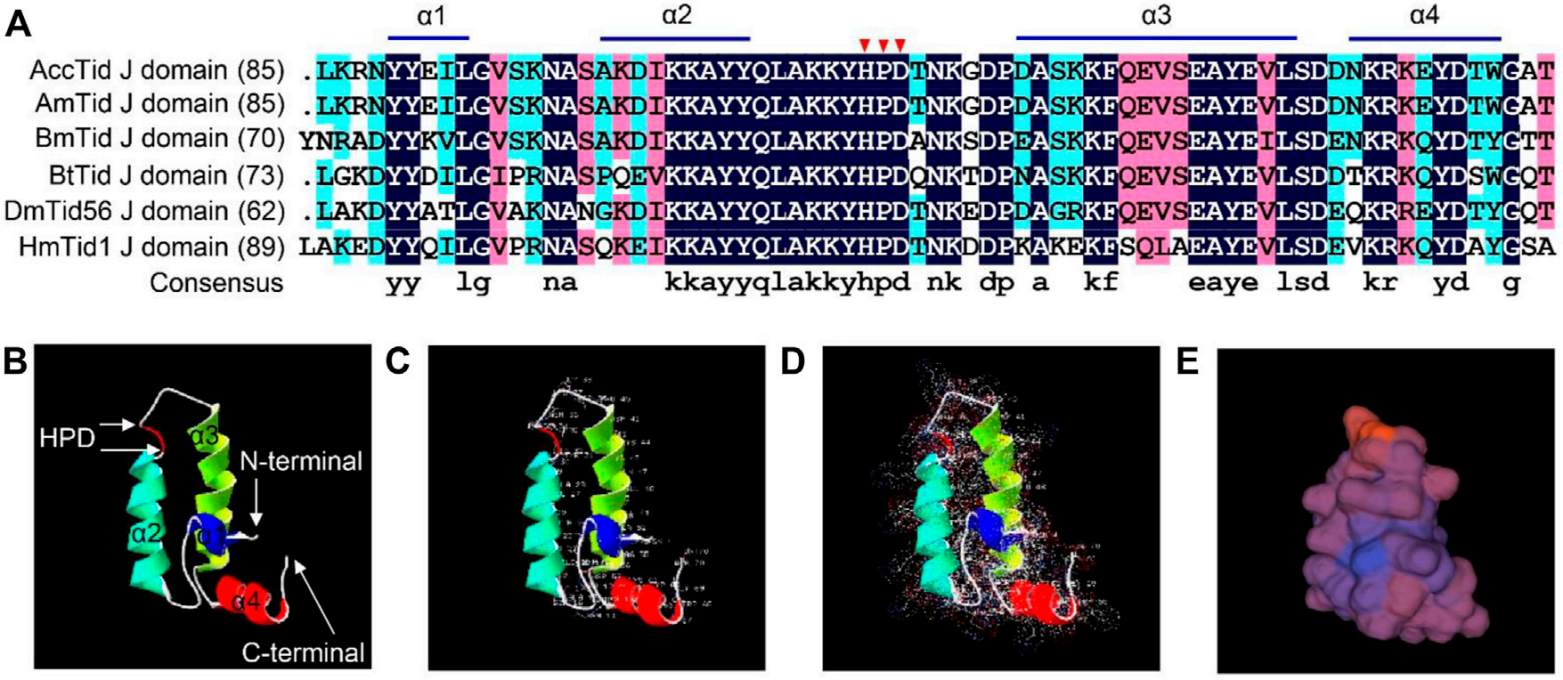
Multiple amino acid alignment and tertiary structure of the Tid J domain. **(A)** Multiple amino acid alignment of the Tid J domains of *A. cerana* and other animals. The HPD motif is indicated by red triangles. AmTid: *Apis mellifera* Tid. BmTid: *Bombyx mori* Tid. BtTid: *Bemisia tabaci* Tid. DmTid: *Drosophila melanogaster* Tid. HmTid: *Homo sapiens* Tid. The protein IDs of AmTid, BmTid, BtTid, DmTid and HmTid are XP_026294788.1, NP_001266353.1, QNN30814.1, NP_995932.1 and NP_001128582.1, respectively. **(B)** Tertiary structure of the AccTid J domain. **(C–E)** show the amino acid residues, electrostatic surface distribution and surface of the AccTid J domain, respectively.

AccTid has a J domain, including four α-helices (helix I, helix II, helix III and helix IV) and a tripeptide composed of His, Pro and Asp (HPD) (Figure 3). The sequence identity of the J domain was high (82.64%) among *A. cerana*, *A. mellifera*, *Bombyx mori*, *Bemisia tabaci*, *Drosophila melanogaster* and *Homo sapiens* (Figure 3A). Tertiary structure analysis showed that the HPD tripeptide was located in the loop between helix III and helix II (Figures 3B–E). These results suggest that AccTid is a typical DnaJ protein.

### Expression profile of *AccTid* under heat stress

**FIGURE 4:**
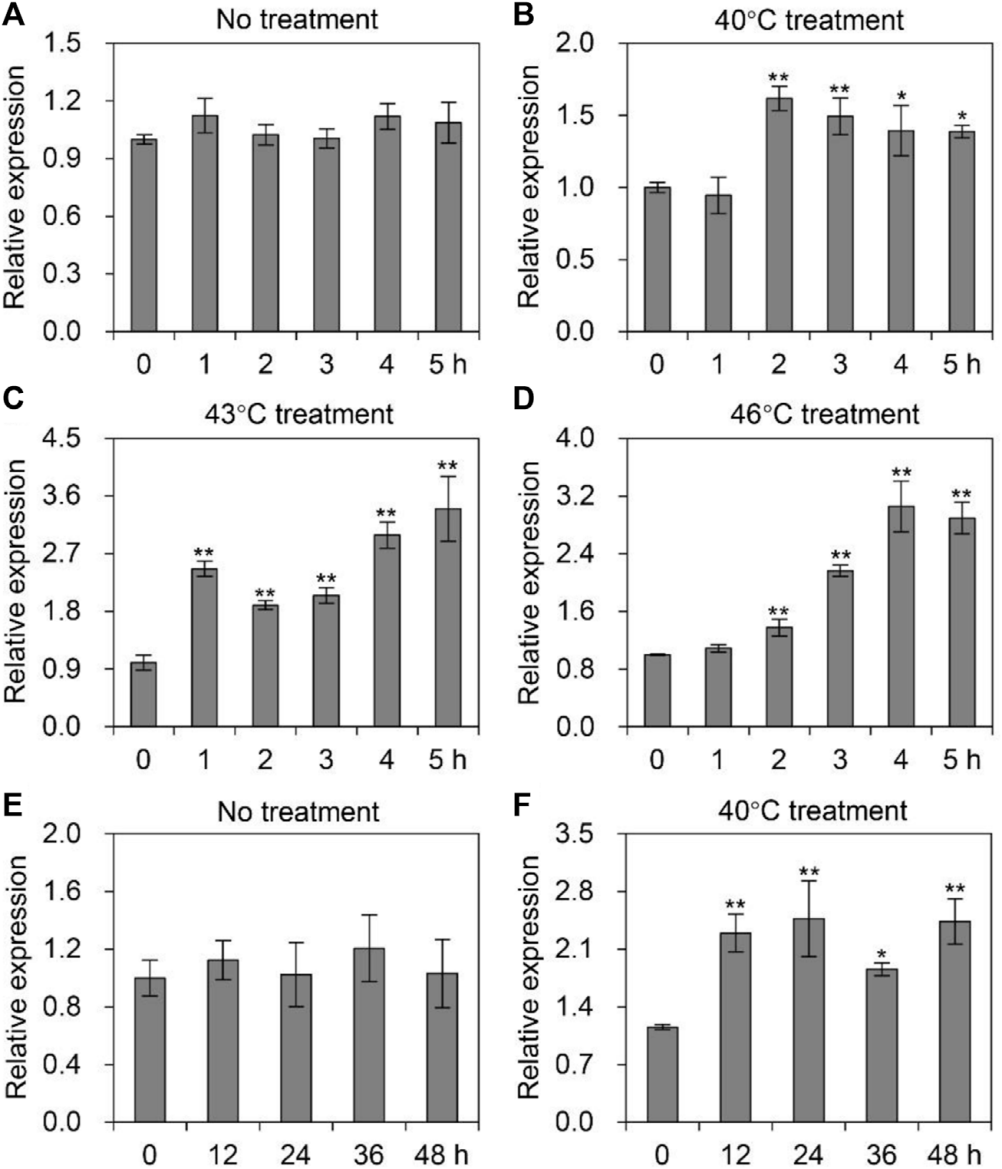
Expression levels of *AccTid* under heat stress. The mRNA levels of *AccTid* under treatment at 40°C **(B)**, 43°C **(C)** and 46°C **(D)** for 5 h relative to those under 33°C conditions **(A)**, and under 40°C treatment **(E)** for 48 h relative to those under 33°C conditions **(F)**. Significant differences were indicated by *p < .05 and **p < .01 according to Bonferroni’s test. *β-actin* was used as an internal control.

To detect the transcription level of *AccTid* under heat stress, honeybees were exposed to different heat gradient (40°C, 43°C, and 46°C) treatments for 5 h. The expression pattern of *AccTid* showed no significant change at 33°C (Figure 4A). When *A. cerana* was exposed to temperatures of 40°C, 43°C, and 46°C, the mRNA levels of *AccTid* were increased relative to the control, and the degree of induction was higher at 43°C and 46°C than at 40°C. The *AccTid* transcripts reached their highest levels at 2 h, 5 h, and 4 h under 40°C, 43°C, and 46°C treatment, respectively (Figures 4B–D). To further investigate the role of *AccTid* in response to heat stress, we prolonged the treatment time to 48 h. There was no significant change in the *AccTid* expression level at 33°C after 48 h of treatment (Figure 4E). However, when *A. cerana* was exposed to 40°C, the expression profile of *AccTid* was continuously induced from 12 h to 48 h (Figure 4F). These results indicate that the expression of *AccTid* plays an important role not only under heat stress.

### Transcriptional level of *AccTid* in response to agrochemicals

**FIGURE 5:**
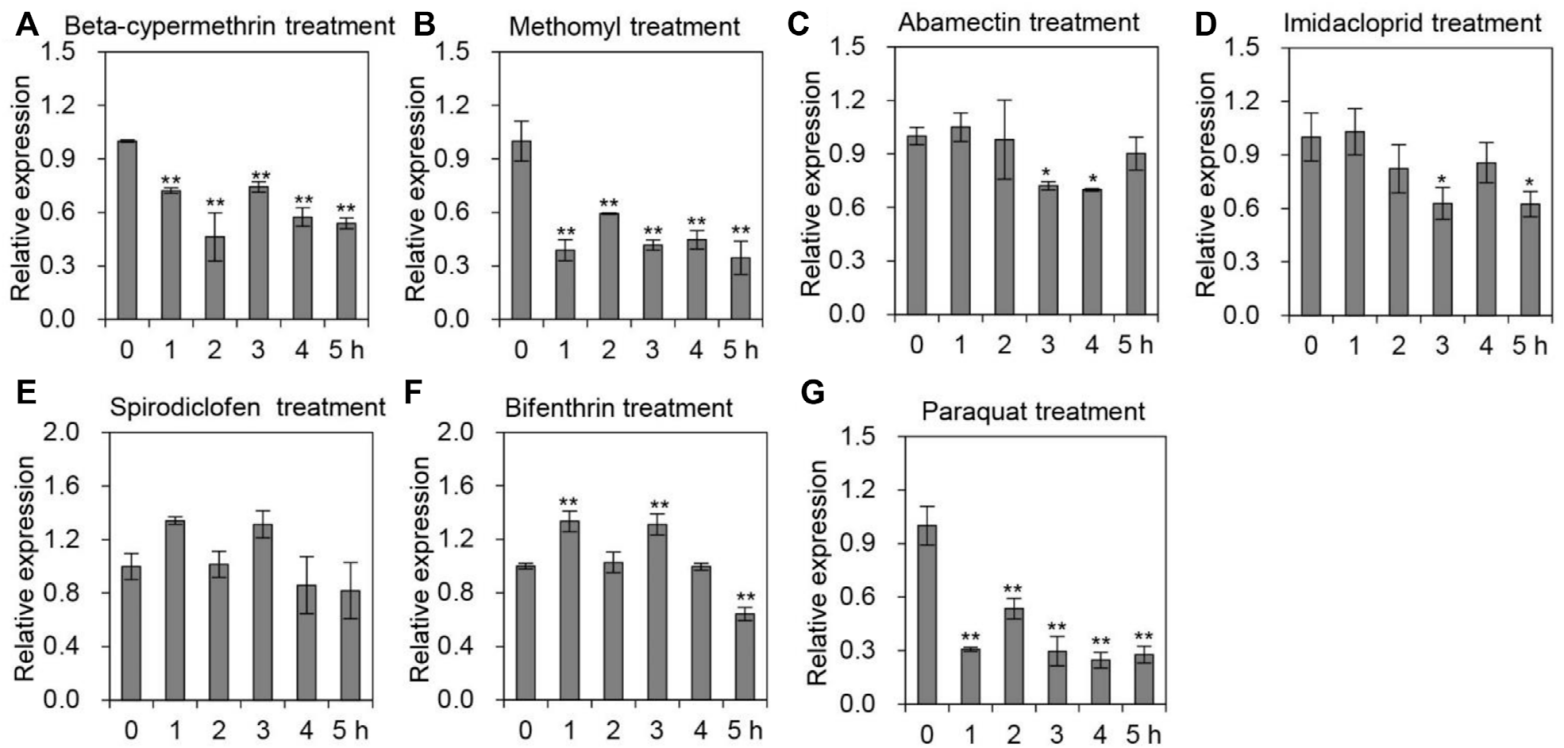
Transcriptional levels of *AccTid* under different pesticide stresses. The transcriptional levels of *Tid* when *A. cerana* was exposed to beta-cypermethrin **(A)**, methomyl **(B)**, abamectin **(C)**, imidacloprid **(D)**, spirodiclofen **(E)**, bifenthrin **(F)** and paraquat **(G)** stress. The internal control was *β-actin*, and *p < .05 and **p < .01 suggest significant differences according to Bonferroni’s test.

To explore whether *AccTid* responds to different agrochemical stresses, *A. cerana* was subjected to cypermethrin, methomyl, abamectin, imidacloprid, spirodiclofen and bifenthrin stress. After exposure to cypermethrin, we found that the expression level of *AccTid* was reduced, showing the minimum value at 2 h (Figure 5A). Under methomyl stress conditions, *AccTid* was downregulated from 1 h to 5 h and reached a minimum level at 5 h (Figure 5B). The expression of *AccTid* was inhibited under abamectin stress and reached a minimum level at 4 h (Figure 5C). Under imidacloprid stress, the mRNA level of *AccTid* showed no significant change at 1 h and was then downregulated from 2 h to 5 h compared to the control (Figure 5D). As shown in Figures 5E, F, the transcription level of *AccTid* was slightly increased at 1 h and 3 h under spirodiclofen and bifenthrin stress but was subsequently downregulated from 4 h to 5 h relative to 3 h. Besides, the mRNA level of *AccTid* was inhibited by paraquat stress from 2 h to 5 h (Figure 5G). These results indicate that the response ability of *AccTid* differed under treatment with different agrochemicals.

### Expression profiles of *AccTid* under cold, H_2_O_2_, UV and sodium arsenite treatment stresses

**FIGURE 6:**
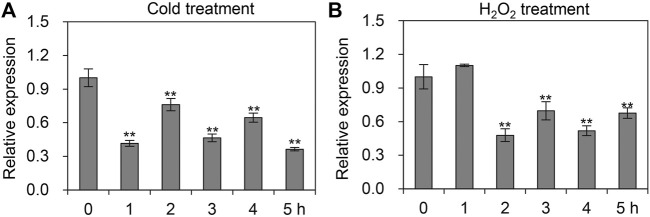
mRNA levels of *AccTid* under cold **(A)** and H_2_O_2_ **(B)**. qRT–PCR was used to measure the mRNA level of *Tid* when *A. cerana* was subjected to cold and H_2_O_2_ stresses. The internal control is *β-actin*, and *p < .05 and **p < .01 suggest significant difference according to Bonferroni’s test.

In addition to various agrochemicals, we also quantified the mRNA levels of *AccTid* in response to cold, H_2_O_2_, UV and sodium arsenite stresses. As presented in Figure 6, the expression pattern of *AccTid* was significantly decreased when *A. cerana* was exposed to cold and H_2_O_2_ stress relative to that in the absence of stress, indicating that *AccTid* is involved in these responses. Under UV and sodium arsenite stress conditions, the transcriptional level of *AccTid* showed no significant change over 5 h (Supplementary Figure S3), which suggested that *AccTid* may not be associated with UV- and sodium arsenite-related activation or that the degree of UV and sodium arsenite exposure in our study may not have been sufficient to alter the expression level of *AccTid*.

### *AccTid* knockdown and the effect of *AccTid* knockdown on the heat tolerance on *A. cerana*

**FIGURE 7:**
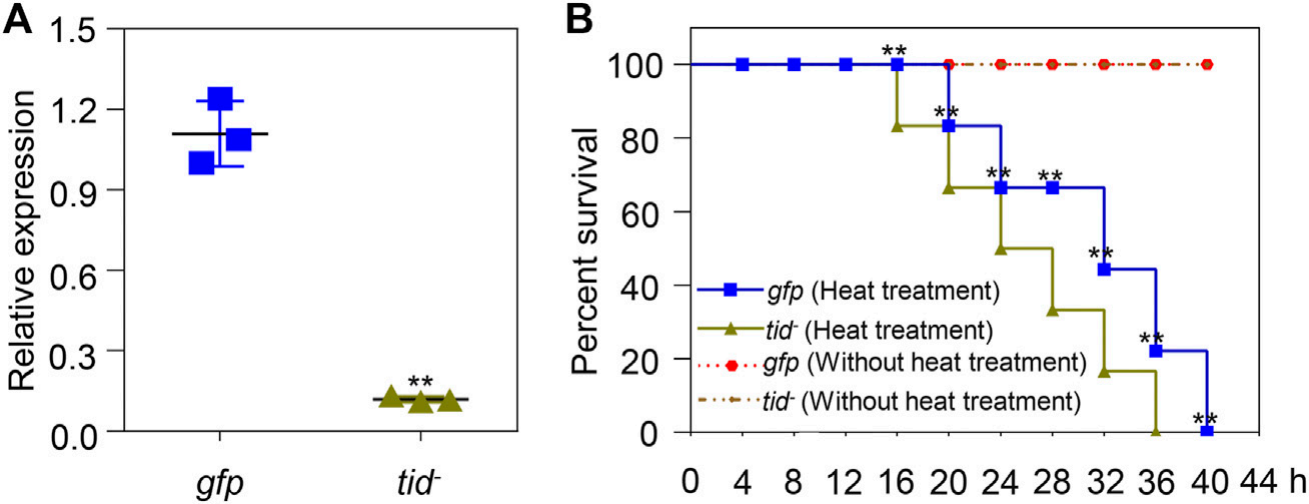
Validation of the efficiency of *AccTid* knockdown **(A)**, and the impact of silencing *AccTid* on honeybee heat tolerance **(B)**. The efficiency of *AccTid* knockdown was detected using qRT–PCR, and *β-actin* was used as an internal control. The abbreviations *tid* ^−^ and *gfp* indicate *AccTid* knockdown and the GFP control, respectively. **p < .01 indicates significant differences according to Student’s *t*-test. Significant differences between *gfp* heat treatment group and *tid* ^−^ heat treatment group in **(B)** are denoted with black **.

To further explore the function of *AccTid* under stress conditions, *AccTid* knockdown was performed using dsRNA-AccTid. In this assay, 19-day-old workers were fed dsRNA-AccTid and dsRNA-GFP, and the efficiency of RNAi was evaluated 1 day postfeeding. Distinct transcriptional suppression was found in the dsRNA-AccTid-fed group relative to the control group fed dsRNA-GFP (Figure 7A), indicating that *AccTid* was successfully silenced. In addition, we found that silencing *AccTid* increased the heat-induced death of honeybees compared with the control group that was fed dsRNA-GFP, and *AccTid* knowckdown by itself is not detrimental to survival (Figure 7B), indicating that *AccTid* plays important roles in the heat stress response.

### Determination of the expression levels of other *Hsps* and antioxidant genes after *AccTid* knockdown

**FIGURE 8:**
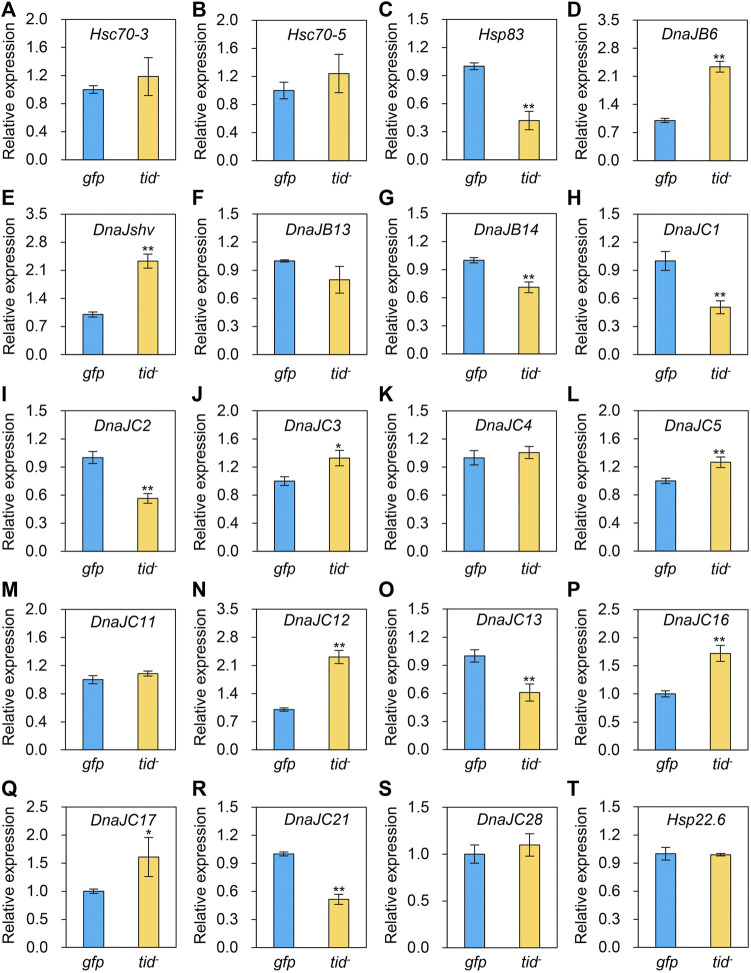
Impacts of *AccTid* knockdown on the mRNA levels of twenty *Hsps* **(A–T)**. *gfp*: GFP control. *tid*-: knockdown of *AccTid*. *β-actin* was used as an internal control. Significant differences between two groups are indicated by *p < .05 and **p < .01 based on Student’s *t*-test.

**FIGURE 9:**
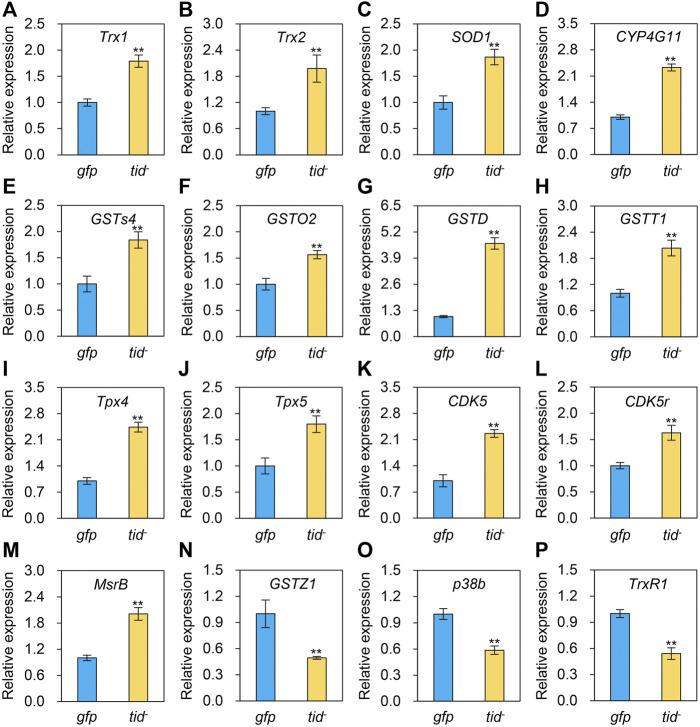
Effects of *AccTid* knockdown on the expression profiles of sixteen antioxidant genes **(A–P)**. *tid-*: knockdown of *AccTid. gfp*: GFP control. Significant differences between two groups are indicated by **p < 0.01 based on Student’s *t*-test. *β-actin* was used as an internal control.

To explore the underlying mechanism of *AccTid* in defence against various stresses, we measured the expression profiles of other *Hsps* and antioxidant genes after *AccTid* knockdown. The mRNA levels of *Hsp83*, *DnaJC1*, *DnaJC2*, *DnaJC13*, *DnaJC21* were decreased, the expression levels of *DnaJB6*, *DnaJshv*, *DnaJC3*, *DnaJC5*, *DnaJC12*, *DnaJC16*, *DnaJC17* were increased, and the transcriptional level of *Hsc70-3*, *Hsc70-5*, *DnaJB13*, *DnaJC4*, *DnaJC11*, *DnaJC28* and *Hsp22.6* has no significant change after *AccTid* knockdown (Figure 8). Besides, the transcriptional levels of many antioxidant genes, including *Trx1*, *Trx2*, *SOD1*, *CYP4G11*, *GSTS4*, *GSTO2*, *GSTD*, *GSTT1*, *Tpx4*, *Tpx5*, *CDK5*, *CDK5r* and *MsrB*, were upregulated when *AccTid* was silenced (Figure 9). However, the mRNA levels of *GSTZ1*, *p38b* and *TrxR1* were downregulated when *AccTid* was knocked down (Figure 9). These findings suggest that *AccTid* influences the expression of antioxidant genes and that the induced *Hsps* and antioxidant genes may be involved in functional compensation for the silencing of *AccTid* in *A. cerana*.

## Discussion

*A. cerana* is a native honeybee in China, where it plays essential roles in pollination services and the economy. However, colony loss of *A. cerana* has often occurred in recent years, partially due to exposure to a wide variety of environmental stresses (Theisen-Jones and Bienefeld, 2016; Chen et al., 2017; Gemeda et al., 2017). Thus, it is important to understand the molecular defence mechanism that *A. cerana* uses to protect itself and reduce the adverse effects of stress factors. Here, we found that *Tid* plays a crucial role in the responses of *A. cerana* to various stresses.

Tid1 in humans, Tid56 in *Drosophila melanogaster* and the Tid proteins of many other animals are homologous proteins (Kurzik-Dumke et al., 1995; Lu et al., 2006; Zhao et al., 2020). There are two isoforms of human Tid1 (Tid1-S and Tid1-L), and the difference between Tid1-S and Tid1-L is the length of their carboxyl-terminal ends (Lu et al., 2006; Banerjee et al., 2022). Consistent with these findings, two isoforms were also found for AccTid (AccTid-L and AccTid-S), and AccTid-L and AccTid-S also differed in their carboxyl-terminal ends (Supplementary Figures S1, S2 and Figure 1). Phylogenetic analysis showed that AccTid was most closely related to AmTid, but AccTid-S was not grouped with AccTid-L (Figure 2), possibly because of the different carboxyl termini of AccTid-L, AccTid-S, AmTid-L and AmTid-S.

The DnaJ protein is defined by the existence of a conserved J domain that comprises four α-helices, and an HPD tripeptide is located in an accessible loop of the J domain (Jiang et al., 2007; Kampinga and Craig, 2010). Human Tid1, also referred to as DnaJA3, has a J domain and belongs to the DnaJA subfamily (Ohtsuka and Hata, 2000b; Qiu et al., 2006). We found that AccTid showed the highest homology with other animal Tid proteins, Tid1 and Tid56, followed by the DnaJA subfamily (Figure 2). In addition, AccTid included a J domain, which contained four α-helices and an HPD tripeptide (Figure 3). These results indicate that AccTid is a typical DnaJ protein and belongs to the DnaJA subfamily.

Human Tid1 plays important roles in cancers, neurodegenerative disorders, differentiation, apoptosis, survival, proliferation, growth and senescence (Banerjee et al., 2022). The functions of Tid56 in *Drosophila melanogaster* and Tid in whiteflies have also been revealed (Kurzik-Dumke et al., 1995; Zhao et al., 2020). However, the function of Tid is not well understood in many animals. In addition, it is worth noting that some DnaJ proteins are related to the heat stress response. For example, the mRNA levels of *DnaJB1*, *DnaJB4* and *DnaJA1* were induced upon heat stress conditions in a T-cell line (Chand et al., 2021). The transcript levels of *DnaJ-1* are significantly upregulated after heat shock in *Drosophila* larvae (Neal et al., 2006). The mRNA and protein levels of *DnaJA1* are induced under heat stress, and *DnaJA1* knockdown decreases the *A. cerana* survival rate (Li et al., 2020). However, whether Tid, as a DnaJ protein, is related to heat stress remains to be elucidated. We found that the expression level of *AccTid* was upregulated under different degrees of heat stress conditions within 5 h or within 48 h, the transcription of *AccTid* was higher at 43°C and 46°C than 40°C from 3 h to 5 h of treatment (Figure 4), and the knockdown of *AccTid* increased the mortality of *A. cerana* under heat stress (Figure 7B). These results indicate that the expression of *AccTid* is important in the response to heat stress and that the requirement of *A. cerana* for *AccTid* depends on the degree and duration of heat treatment.

In addition, we found that the mRNA level of *AccTid* was reduced under treatment with various agrochemicals, such as beta-cypermethrin, methomyl, abamectin, imidacloprid, bifenthrin and paraquat (Figure 5). Agrochemicals are among the drivers that impact honeybee mortality and fitness (Goulson et al., 2015; Siviter et al., 2021). The transcription levels of *AccTid* may be involved in the responses of *A. cerana* to various agrochemicals. Imidacloprid is a neonicotinoid that is a neuroactivator of nicotinic acetylcholine receptors in insects (Palmer et al., 2013). Abamectin is one of the commonly used avermectins, which have neurotoxic impacts, inhibit neurotransmission and target the γ-aminobutyric acid receptor (Mellin et al., 1983; Lasota and Dybas, 1991). As an oxime carbamate pesticide, methomyl restrains the activity of acetylcholinesterase, triggering the failure of nerve tissue function (Silva Filho et al., 2004; Lin et al., 2020). Beta-cypermethrin is a type II synthetic pyrethroid insecticide that primarily acts on potassium, sodium and calcium channels to trigger neurotoxicity (Breckenridge et al., 2009; Cao et al., 2015). As a pyrethroid insecticide, bifenthrin disrupts sodium ion channels and causes neurotoxicity (Soderlund et al., 2002). Therefore, the changes in *AccTid* expression levels under beta-cypermethrin, methomyl, abamectin, imidacloprid and bifenthrin may influence the molecular mechanisms of action of these agrochemicals.

Besided, Many stresses, such as heat, agrochemical and cold stress can trigger oxidative stress and damage in organisms (Houston et al., 2018; Huang et al., 2021). It has been shown that heat stress triggers oxidative DNA damage and increases sperm mitochondrial ROS generation in the male germline (Houston et al., 2018). Imidacloprid impairs movement in the *Procambarus clarkii* digestive system through oxidative stress and neurotoxicity (Huang et al., 2021). Methomyl exposure increases oxidative stress and damages mitochondrial function in mice (He et al., 2021b). Beta-cypermethrin influences reproduction by increasing oxidative stress in the uterine tissue of female mice (Zhou et al., 2018). The generation of oxidative stress and dependence on redox cycling are the main molecular mechanisms of paraquat, which is toxic (Dinis-Oliveira et al., 2008). Cold stress perturbs the balance between the antioxidant and oxidant systems and causes oxidative damage by altering protein oxidation, non-enzymatic and enzymatic antioxidant status, and lipid peroxidation in male Wistar rats (Sahin and Gumuslu, 2004). The expression level of *AccTid* was shown to be altered under heat, cold, H_2_O_2_, paraquat and some agrochemical stresses (Figure 4-Figure 6). In addition, we found that the transcriptional levels of some antioxidant genes were upregulated after the knockdown of *AccTid* (Figure 9). These findings suggest that *AccTid* functions in oxidative damage triggered by stressors by indirectly influencing antioxidant genes, and the upregulation of these antioxidant genes may be a functional complement of *AccTid* knockdown.

In addition, as a cochaperone, DnaJ is functionally connected with the Hsp70 family (Hennessy et al., 2000; Kampinga and Craig, 2010). We found that the expression levels of two Hsp70 genes, *Hsc70-3* and *Hsc70-5*, showed no significant changes after *AccTid* knockdown (Figures 8A, B), indicating that the role of *AccTid* in the stress response is not related to *Hsc70-3* and *Hsc70-5*. However, when *AccTid* was knocked down, the mRNA levels of some *DnaJs,* including *DnaJB6*, *DnaJshv*, *DnaJC3*, *DnaJC5*, *DnaJC12*, *DnaJC16*, and *DnaJC17,* were increased (Figure 8), which may contribute to honeybee defence against stress after *AccTid* knockdown.

In conclusion, our results showed that *AccTid* is a typical DnaJ protein. The responses of *AccTid* to different stress conditions show obvious differences, which may be a strategy for coping with multiple stress factors. The knockdown of *AccTid* influenced the survival rate of *A. cerana*, and the transcriptional levels of some *Hsps* and antioxidant genes, indicating that *AccTid* may play vital roles under stress conditions by directly or indirectly influencing the expression of antioxidant genes in *A. cerana*. These results provide insights into the function of *Tid* in the stress response and enrich the understanding of the *A. cerana* defence mechanism under environmental stresses.

## Data Availability

The datasets presented in this study can be found in online repositories. The names of the repository/repositories and accession number(s) can be found in the article/Supplementary Material.
