# Supplementary figures and images for: Characterisation of the heat shock protein Tid and its involvement in stress response regulation in Apis cerana
Source: Front Physiol. 2022 Dec 22;13:1068873. doi: 10.3389/fphys.2022.1068873 (PMC9813389; doi:10.3389/fphys.2022.1068873)

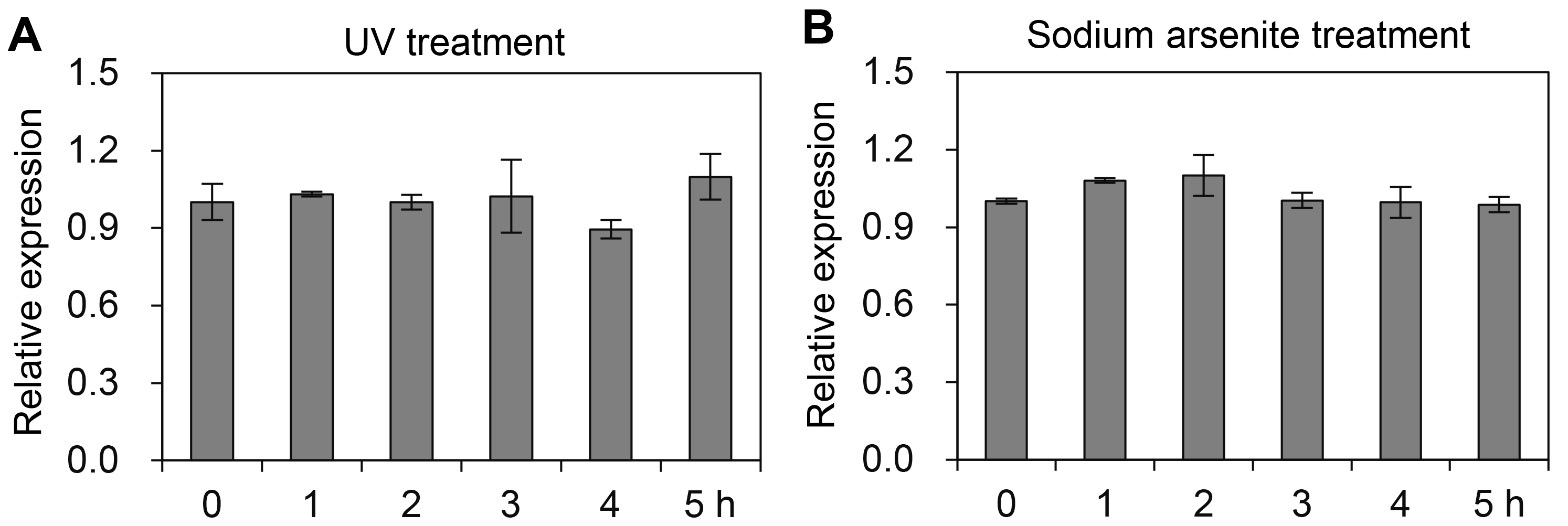

Supplement: Supplementary file 1 [file Image3.JPEG]

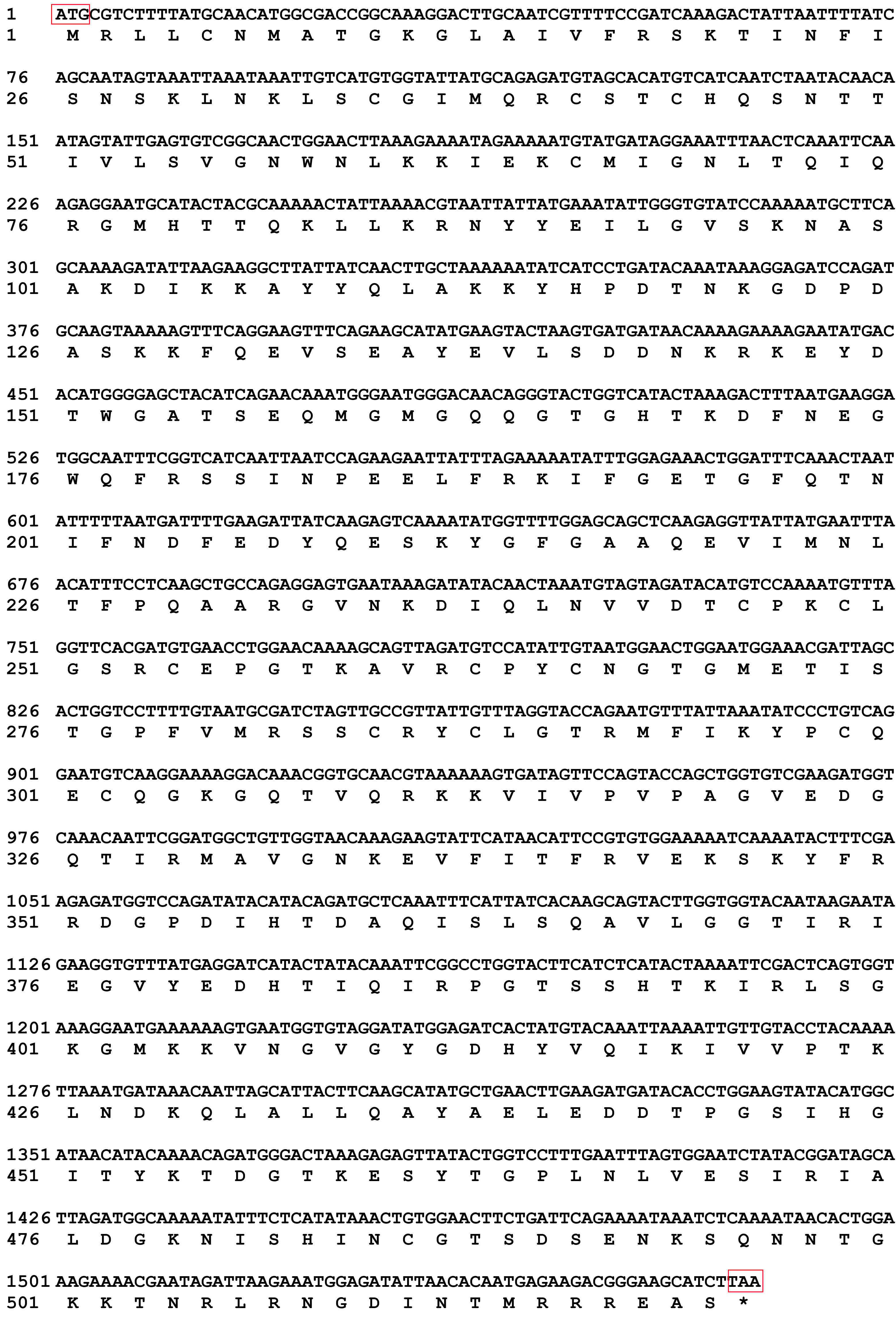

Supplement: Supplementary file 2 [file Image1.JPEG]

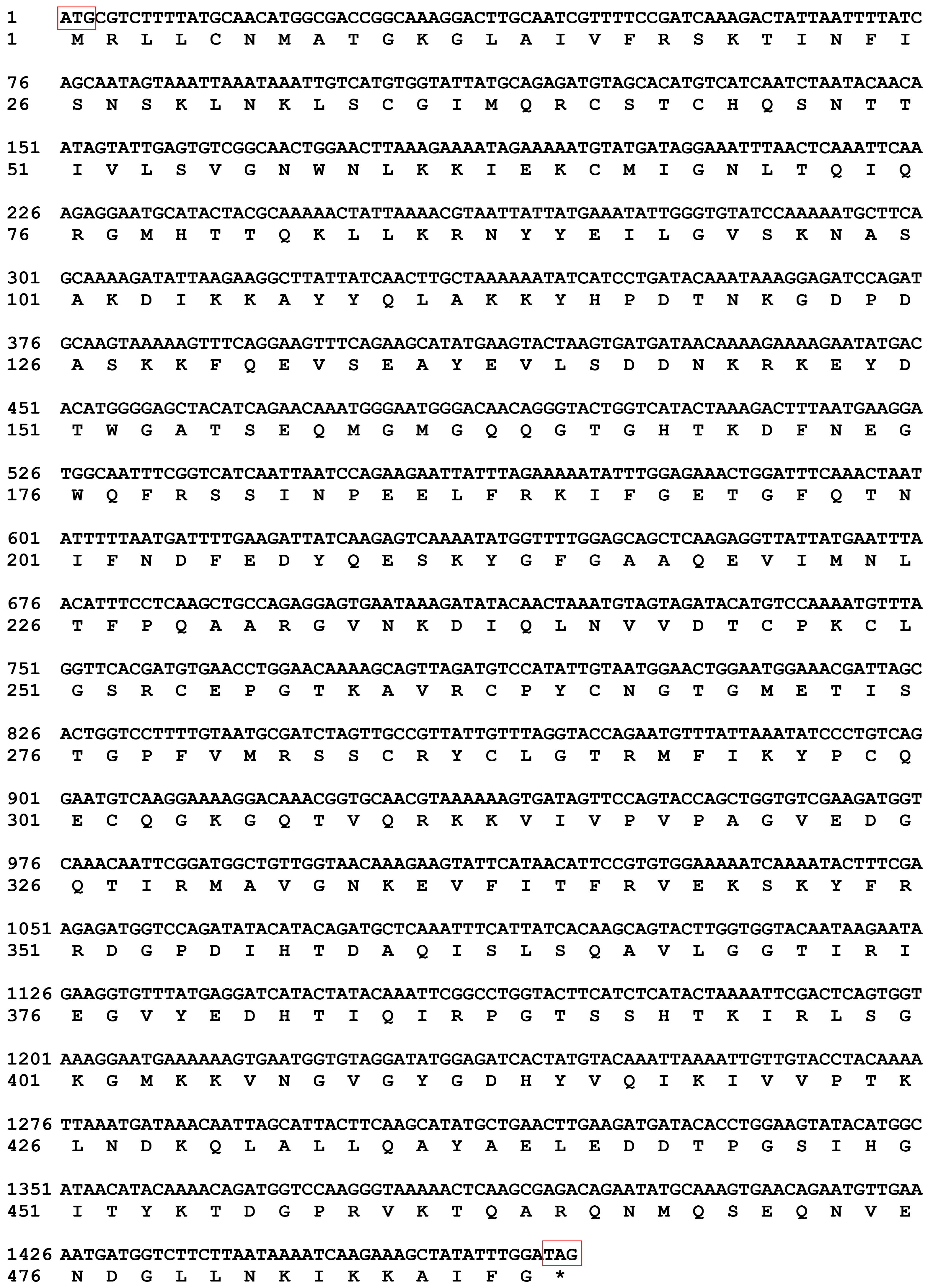

Supplement: Supplementary file 3 [file Image2.JPEG]
